# Long-term home visiting with vulnerable young mothers: an interpretive description of the impact on public health nurses

**DOI:** 10.1186/s12912-015-0061-2

**Published:** 2015-03-08

**Authors:** Anne L Dmytryshyn, Susan M Jack, Marilyn Ballantyne, Olive Wahoush, Harriet L MacMillan

**Affiliations:** Stollery Children’s Hospital, 8440-112 St, Edmonton, Alberta T6G 2B7 Canada; School of Nursing, McMaster University, 1280 Main St. West, Hamilton, Ontario L8S 4 K1 Canada; Holland Bloorview Kids Rehabilitation Hospital, 2W305, 150 Kilgour Road, Toronto, Ontario M4G 1R8 Canada; Departments of Psychiatry and Behavioural Neurosciences and Pediatrics, Offord Centre for Child Studies, McMaster University, Wilcox Building, 1280 Main St. West, Hamilton, ON L8S 4 K1 Canada

**Keywords:** Public health nursing, Home visiting, Compassion fatigue, Burnout, Compassion fatigue, Compassion satisfaction, Workplace factors

## Abstract

**Background:**

The Nurse-Family Partnership (NFP) is a targeted, nurse home visitation program for young, low-income, first-time mothers. While the effectiveness of the NFP has been established in the United States, and is currently being evaluated in the Canadian public health care system, we have minimal understanding of how work of this nature impacts public health nurses (PHNs), an essential component of this program delivery model, on both professional and personal levels.

**Methods:**

This two-phase study consisted of a qualitative secondary analysis of data from five focus groups conducted with PHNs (N = 6) who delivered the NFP intervention as part of a pilot study assessing feasibility and acceptability conducted in Hamilton, Ontario. The second phase, an interpretive description of individual interviews with the PHNs (N = 10) who have delivered the NFP in this context, further explored themes identified in the first phase. A practice, problem and needs analysis was conducted to describe and understand the phenomenon and promote sustainability of PHNs in this practice environment. Conventional content analysis was used to code and categorize data in the two datasets.

**Results:**

The nurse-client relationship, the core elements and structure of the NFP program and support of NFP colleagues were described as rewarding factors, while workload and workplace factors were identified as significant contributors to stress. PHNs described transforming their nursing practice through redefining success and shifting to a philosophy where the client is the expert of her own life. PHNs described the personal impact of worry about clients and doubt about their effectiveness in addressing client concerns. High levels of satisfaction were described in relation to the depth and intensity of relationships with clients and seeing them succeed over time.

**Conclusions:**

PHNs are impacted in multiple ways by their work with vulnerable, young mothers. The study findings have implications for identification of strategies to support PHNs in reducing staff turnover, PHN burnout, secondary traumatic stress and compassion fatigue, and improving program delivery.

## Background

Compared to other forms of employment, the delivery of patient care has the potential to impact the physical and emotional health of the health care provider. A recent Canadian study involving 19,000 nurses reported that nurses experience a higher rate of several chronic health conditions compared to the general population of employed Canadians and two-thirds reported that their workload was too much for one person [1].

Although nurses work in a wide variety of settings, the majority of research has focused on work-related professional and personal consequences for hospital-based nurses who practice in specialty areas such as oncology, intensive care, or pediatrics [2-5]. To date, there is limited research focusing on the potential impacts and consequences for nurses who practice in community-based settings. Given the varying complexity of community or home-based nursing roles and the populations they serve, the challenges and consequences may differ from the findings associated with hospital-based nursing practice.

Delivery of health services to families with young children has a long tradition within public health nursing [6]. Many home visiting programs focus service provision on vulnerable members who have less access to financial resources and social support such as young, socially disadvantaged mothers and their infants [7-10]. These families are at risk for a range of health problems including substance abuse and poor mental or physical health. In addition, intimate partner violence (IPV) and knowledge or skill deficits around parenting can result in a higher incidence of child maltreatment or neglect. Home visiting programs can facilitate access to families who are less likely to seek out medical care or use early intervention services [8].

A strong therapeutic relationship between the health professional and the mother is essential to the success of home visiting programs [11-14] however the formation of this relationship through empathic engagement may also precipitate work-related stress [15]. Poverty and complex cyclical familial issues are only some of the overwhelming range of issues facing mothers on a daily basis; and the complexity and chronicity of issues in the lives of clients often stands in stark contrast to the public health nurses’ (PHN) own experiences in their typically middle-class backgrounds [16].

One nurse home visitation program is the Nurse-Family Partnership (NFP), an evidence-based, primary prevention intervention for young, low-income, first time mothers [17]. The three overarching goals of this program are to: 1) improve health-related behaviours during pregnancy; 2) improve parenting skills to promote child health and development; and 3) assist parents to attain employment and meet educational goals, and plan for future children [18]. Women are enrolled in the program by the end of the 28^th^ week of pregnancy and home visits continue until the child’s second birthday. PHNs follow a schedule of home visits that vary throughout the program (weekly, biweekly or monthly) [17,19].

The NFP program has been rigorously evaluated in the United States (US) and has demonstrated consistent effects across three randomized controlled trials (RCT), including improved prenatal health, a reduction in childhood injuries, fewer subsequent pregnancies with increased intervals between births, and improvements in maternal economic self-efficacy [9,10,17,18,20,21]. Given this success, there has been extensive international interest in evaluating and implementing the NFP beyond the US. The Prevention Research Center for Family and Child Health, University of Colorado Denver has developed a four-step international replication process: 1) adaptation of the NFP materials to the new context; 2) piloting the NFP for acceptability and feasibility; 3) conduct of a RCT to determine effectiveness of the NFP compared to locally delivered services; and 4) broader implementation [22]. In 2008, through a partnership between McMaster University and Hamilton Public Health Services, the first pilot of the NFP in a Canadian context was initiated in Hamilton, Ontario [23].

Given their educational background, knowledge of maternal and child care and expertise in forming therapeutic and empathetic relationships, PHNs are ideally suited to deliver the NFP. While there is evidence available supporting the effectiveness of the NFP and describing challenges of nursing, there is little understanding of the PHNs’ experiences of delivering an intense, and largely relationship-based, long-term home visiting program. Although Zeanah, Larrieu, Boris and Nagle [24] provided an account of American NFP PHNs perspectives, their study only included experienced PHNs and focused on the challenges of addressing client’s mental health issues.

Although the NFP has a structured program-delivery model, in essence, the PHN *is* the intervention. They are the sole provider of program content and must continually balance between addressing emerging family issues and material in the visit-to-visit guidelines [25]. Given the emphasis placed on the importance of this relationship for the success of the program, it is interesting that McNaughton observed in her review of 13 nurse home visiting programs [26], no studies reported on how this nurse-client relationship was developed or noted to be successful. Although literature on the establishment of the nurse-client relationship in home visiting is substantial [11,14,27,28] this crucial component is often glossed over when reporting program outcomes. Impacts on PHNs involved in long-term home visiting have not been well described.

The purpose of this study was to identify and describe the nature of the challenges and perceived benefits experienced by all PHNs working in the NFP program in Hamilton, Ontario, Canada and to explore what was needed to effectively deliver the program. Strategies are needed to support the PHNs who deliver important and successful programs like the NFP, however current literature does not capture the unique challenges of nurse home visiting with a complex population of high-risk, young mothers in their home environment. For an intervention to be meaningful and effective the nature of the problem, what providers need and what is currently being done in the practice environment must be described.

## Methods

This qualitative interpretive descriptive study was conducted in two distinct phases. The first phase was a secondary analysis of five 90–120 minute focus groups conducted with PHNs (N = 6) who delivered the NFP intervention as part of a pilot study assessing the feasibility and acceptability of the NFP conducted in Hamilton, Ontario between 2009–2011 (for additional details about the methods see Jack et al. [23]). Themes related to the PHNs experience of delivering the NFP were captured. The focus groups were facilitated by a nurse-researcher (SMJ) with extensive experience in qualitative research. In the pilot study, study questions focused on the acceptability of the NFP model, nurse/supervisor education, PHN role, feasibility of the NFP program in the local context, as well as challenges and benefits of delivering the NFP on both a personal and professional level.

Themes identified from the focus group data analysis were explored or clarified in the second phase: single, 60–90 minute individual, semi-structured interviews with the initial PHNs (N = 6) and NFP PHNs (N = 4) who were hired by Hamilton Public Health Services after the pilot study was completed. The interviews were completed by a nurse-researcher (AD) who has a rich clinical background in maternal/child health and professional nursing practice issues. After obtaining written, informed consent, interviews were recorded and conducted in a private location selected by the PHN at a mutually convenient time. As all of the past and current NFP PHNs (N = 10) consented to participate, no further sampling strategy was necessary. The only inclusion criterion was to be a PHN who participated in the Hamilton NFP. The semi-structured interview guide (summary provided in Table 1) was developed from themes identified in the first phase (Table 2), specifically those related to: the nurse-client relationship, challenges and impact of delivering the NFP on PHNs, and formal and informal support mechanisms for nurses. The structure of the questions were informed by the initial two stages of van Meijel, Gamel, van Swieten-Duijfjes & Grypdonck’s framework for using qualitative methods to develop interventions [29]. In these stages, the problem is defined and a problem, needs and practice analysis is conducted [29]. Demographic information was collected. Individual interviews ensured that experiences from each PHN were heard and also allowed for a deeper exploration of identified themes from the first phase. Field notes were maintained by all researchers responsible for the collection of the primary data (SJ, AD). The content of the field notes included observational descriptions of the interviews and the contexts in which they occurred, participants’ reactions and non-verbal language in the interviews and notations of emerging themes. The information in the field notes informed the study audit trails, the development of additional questions to add to the interviews and created a space to document emerging interpretations of the data. A reflective journal was also kept to capture changing ideas or personal responses to the ongoing research [30]. This study was reviewed and approved by the Hamilton Integrated Research Ethics Board.

**Table 1:** **Summary of Semi-Structured Interview Guide Questions**

**Primary questions**	**Examples of probes**
The focus group discussions provided a picture of the challenges of working in the NFP but also a love for the work. What is it about delivering the NFP that you find personally rewarding?	a. The strength and value of the relationship between PHNs and young mothers was a theme that clearly surfaced in the focus groups. What has been your experience of this relationship? Why is this relationship important?
	b. What specifically about the NFP program facilitated the development of this relationship?
	c. What are the benefits of developing such a powerful relationship with your clients?
	d. What are the potential difficulties?
	e. NFP nurses have shifted away from a perspective of “doing to” their clients, recognizing that the client is the expert of her own life. How would you describe this new perspective? How does this compare with other health care/social service providers approach?
“What are the most significant challenges you experience in delivering the NFP program with fidelity to the model elements?”	a. Probe for challenges at different levels: client issues, interpersonal (nurse-client relationship), NFP team/within the organization, community.
	b. How do the challenges change throughout the phase of the program?
	c. How do you find these challenges affect you?
	d. How do you respond to these challenges?
	e. Are your current strategies effective? Why or why not?
	f. How do you reconcile the passion for the program with the personal toll?
	g. What do you need from your workplace to support you in this role?
	h. What could be improved?
	i. What is the value of reflection in your nursing practice?
	j. How does (take responses from above) influence your professional nursing practice and your ability to deliver the NFP?

**Table 2:** **Summary of Findings from Phase 1 Focus Groups**

**Category**	**Sub-category**	**Codes**
PHN perceptions of the NFP	Evidence of program effectiveness	
	Program structure and guidelines	
	Frequency and intensity of home visits	
Relationships in the NFP	PHN-client relationship	Time, and timing, to build the relationship
		Barriers to the relationship
		• Trust issues
		• Client fatigue
		• Transient/unstable housing
		• Changes in clients relationship status
		• Scheduling conflicts/overload
		• Client withdrawal
		• Mental health concerns
		Maintaining the relationship
		• Being there
		• Consistency
		PHN transformation in the NFP
		Client as expert
	Changing relationship with other service providers	
Delivering the NFP: Challenges and impact on the PHN	Professional and work-related challenges	Challenges
		• Confidentiality of NFP content
		• Workspace and resource issues
		• Theoretical differences from other service providers
		• Demands of a full case load
		• Sense of overload
		• Documentation
		• Time in the car
		• Accessing satellite offices
		• Scheduling realities of NFP clients
	Personal impacts	Sense of doubt
		Impact on life outside of work
		Realities of a high risk population
		Emotional impact
		Graduation
		Changing definition of success
		Rewards of delivering the NFP
Formal and informal support mechanisms in the NFP	Team meetings and case conferences	
	Reflective Supervision	Supervision focus
		Time constraints
		Barriers to effective supervision
	Informal peer debriefing	
	Individual stress management practices	
	Need for additional support	

Focus group transcripts were obtained and interview data were transcribed verbatim with identifying information removed. Transcripts were read in their entirety and NVivo9 [31] was used for open coding. A codebook, with definitions for each code, was developed and applied to all transcripts. Conventional content analysis [32] was used to identify themes expressed by the PHNs in their home visiting practice with vulnerable families. In this manner of conventional content analysis a primarily inductive approach was taken where open codes were developed based on concepts/words within the text. In the second stage of analysis, codes were collapsed into categories. Then using a process of constant comparison, categories from all of the data sets were compared for the purpose of identifying the predominant themes.

As data analysis and collection occur concurrently [30] in the second phase, there was flexibility to alter or deviate from the interview guide as themes reached saturation. This allowed more time for an increased depth of discussion to reflect this evolving understanding of the phenomenon. Member checking was conducted by sharing a written synthesis of the study data with PHNs and an invitation to comment on the accuracy of the findings. Participants were also invited to participate in a follow-up phone call.

Interpretive description, a method that extends beyond being simply a qualitative description to include both a practice goal as well as generating an understanding of both what is known and not known about a given area [30] was used in phase two. Within this study, the practice goal was to understand the NFP PHN experience to provide a platform from which approaches to support them could be developed. Interpretive description describes a phenomenon and places it back in its context, with all its nuances and influences without presuming to provide an explanatory model [33].

Overall trustworthiness of the data was enhanced by the application of multiple strategies to address credibility, dependability, confirmability and transferability. Confidence in the credibility of these data as an accurate reflection of Canadian PHNs’ experiences and impact of providing home visits to vulnerable NFP clients was achieved through application of data triangulation, researcher credibility and member checking. To promote overall researcher credibility, the researcher was immersed in the public health PHN environment through an advanced clinical placement. Daily interaction with PHNs, observation of home visits and attendance at team meetings helped to establish rapport and allow for an appreciation of their day-to-day professional life.

## Results

The phase one sample consisted of the total population (N = 6) of PHNs who participated in the NFP pilot study in Hamilton, Ontario. For phase two, the total population of past and current NFP PHNs in Hamilton (N = 10) consented to participate in semi-structured individual interviews providing a 100% response rate. At the time of the interviews, PHNs were an average age of 42 years (range 26–66). Participants had an average of 18 years (range 4–41) of nursing experience and an average of 10 years (range 1.5-30) of nursing in public health. All PHNs had previous home visiting experience with the existing provincial long-term home-visiting program representing an average of 4 years (range 0.2 -12). PHNs had on average enrolled 24 clients (range 9–50) and those from the pilot group (N = 6) had the experience of graduating an average of 12 clients (range 4–15) each. The remaining four PHNs had active caseloads but had not yet graduated any clients. PHNs had a minimum of a bachelor’s degree in nursing and one PHN was pursuing graduate studies.

The key findings from the Phase 1 focus groups are summarized in Table 2, followed by a summary of themes that emerged from the Phase 2 semi-structured interviews in Table 3. The following text presents a synthesis and triangulation of the findings from both phases to highlight the dominant themes and sub-themes of the PHN’s experience in the NFP. The study findings will be outlined in the following themes: 1) PHN’s perceptions of NFP; 2) Centrality of the PHN-client relationship; 3) Impact of the NFP on professional practice; 4) Challenges related to program delivery; and finally, 5) Strategies to support PHNs’ NFP home visitation work.

**Table 3:** **Summary of Themes from Individual Interviews with PHNs**

**Category**	**Area of analysis**	**Sub-categories**
Delivering the NFP	Practice	PHN education
		Home visiting
		Documentation
		Team meetings/case conferences, reflective supervision
	Problem	Lack of community
		Workload and time demands
		• Scheduling
		• Driving time
		• Documentation
		• Team meetings/case conferences, reflective supervision
	Needs	Time Consistent orientation & formal peer support/mentoring
		1Ongoing professional development & education
		Increase in workplace efficiency (minimize driving time, efficient documentation system, dedicated administrative support, improved efficiency of team meetings/case conferences, safety in reflective supervision)
Transition to NFP PHN role	Individual PHN practice	Building the therapeutic relationship
		Redefining success
		Shifting to client as expert
		Using the NFP education
	Problem	Concern for clients
		Working up to a visit/nothing left to give
		Impact of doubt/did I do enough?
		Emotional & physical impact
		Client Graduation
	Needs	(see support section)
Support in the NFP	Individual PHN -practice	Satisfaction in the NFP
		• Therapeutic relationship
		• Making a difference
		• Learning from clients
		Individual coping strategies
		• Boundary setting with clients
		• Reflecting on practice
		• Managing self-expectations & letting go
		• NFP evidence as support
		• Engaging in self-care activities
	NFP program support - practice	Informal peer debriefing
	Problem	Burden of peer debriefing
	Needs	Culture of safety
		Validation and formal preceptor

### PHNs’ Perceptions of NFP

PHNs stated that they were empowered and felt excited to embark on delivering the NFP, an evidence-based program with proven outcomes with a population of vulnerable clients. According to comments expressed by PHNs, the NFP model and approach fit with this specific population, and a structured visitation schedule with visit-to-visit guidelines addressing content over six domains (personal health, environmental health, life course development, maternal role, family and friends, and health and human services) provided a consistent approach to practice. The PHNs appreciated the flexibility to adapt the home visiting materials to meet the needs of their clients, which reflected the reality of forming therapeutic relationships with this population of vulnerable mothers. While many of the NFP program structures, such as the time of program initiation and frequency and duration of home visits, were viewed favourably by the PHNs, the overwhelming strength of the NFP was the importance placed on the therapeutic relationship between PHN and client. In addition, rather than discharge clients who missed appointments, PHNs were able to take the time to locate “hard-to-find” clients, not as an “extra” activity but as an expectation.

PHNs described enjoying coming to work, and loving their job and as one stated, the NFP was “so much more than a job” (PHN FG-03). Multiple factors, from the design of the program to client success stories were reported as fuelling this sense of job satisfaction. PHNs reported positive outcomes with their clients, such as healthy infant attachment, increased rates of breastfeeding, decreased smoking rates and clients returning to work or school. Forming relationships with individual clients, learning from them and seeing improvement over time were outcomes described by the PHNs as maintaining their motivation and engagement in the program. As one PHN explained, she found small gestures, such as being allowed into a client’s home or hearing that clients looked forward to her visits, particularly rewarding. Even when discussing the many challenges, PHNs were also careful to distinguish between challenging circumstances and their client. Often, it was the clients’ situations and living circumstances, rather than the clients that PHNs found stressful.

### Centrality of the PHN-Client Relationship

The long-term nature of the PHN-client relationship allowed for a depth of understanding and connection that was not possible in existing home visitation programs and created the foundation for client retention in the program. Maintaining this relationship over time however, was a challenge. Barriers to relationship formation ranged from the complex, such as a client’s traumatic past and inability to form trusting relationships, to the simple, such as scheduling. Other barriers included client fatigue after the birth of the child, transient or unstable housing, changes in the clients’ intimate partner relationship status as well as the client returning to work or school. Although some clients may “disappear” for several months, the strength of the established relationship in the early period and the proven history of “being there” allowed clients to re-engage with their PHN at a time of their choosing. Although simply “being there” - being a consistent and trustworthy presence in the client’s life was identified as an important factor, PHNs also described skillfully navigating the relationship within the context of the client’s changing circumstances. As one PHN explained:

I think this program has allowed me to be more *flexible* to… the human condition as far as *realistic expectations*… she doesn’t want to talk to me. And that’s *ok*. I’m giving her that chance to not to talk to me and that doesn’t mean I’m going anywhere,… you can *back off* and it’s ok and there’s no penalty to *me* and there’s no penalty to the client. (PHN FG-04)

### Impact of NFP on Professional Practice

The opportunity to deliver nursing care in the NFP, with its focus on early prenatal engagement, frequent home visits, in-depth nursing assessments, education to provide comprehensive, tailored nursing interventions to a targeted population of the most vulnerable populations of families significantly impacted the PHNs. Compared to their past home visiting experiences, PHNs identified that they were “transformed” and were finally given the opportunity to practice nursing at the full scope of their professional practice.

#### PHN transformation

PHNs described experiencing a growth in professional capacity and stated they were finally doing the nursing work they desired and providing care to clients they previously had been unable to reach. One PHN, who described herself as “older,” reflected on the state of nursing before and after the NFP:

Until working in Nurse-Family Partnership I think I really was struggling with the *role* and so now I see my role and I’m saying, ‘*yes* it’s a good role and *yes* the nurse should be in there and *yes* we have something to offer to the community even though other factions have a different philosophy.’ I feel *stronger* and *believe stronger* in my Nurse-Family Partnership philosophies and *theories*. (PHN FG-01)

From a professional practice perspective, the PHNs felt that the NFP education, underlying theories and nursing competencies fundamentally changed the way in which they approached client care and services. Of primary importance was the focus on nursing skills to promote client self-efficacy, compared to past practices where PHNS may have just shared information or provided health education. Reflecting on their experiences delivering the NFP, PHNs discussed overt and subtle shifts in their individual professional nursing practice through a shift to viewing the client as the expert of her own life. With the philosophical underpinnings of the program being driven in part by self-efficacy theory, the PHNs encouraged clients to make their own decisions and take control of their lives. For some PHNs, letting go of this control was challenging yet once this was accomplished, it brought a deeper sense of connection with the client.

#### Redefining success

PHNs were unanimous in their experience of redefining the meaning of success for their clients. Given the variety of clients’ circumstances and histories, success had to be carefully considered for each client and her individual context and PHNs challenged their own assumptions or expectations about what to expect from their clients. As this PHN stated, “We *expect* them to be able to do so much more like what you and I could do and they *can’t*” (PHN 002). Another experienced PHN shared how her perspective changed over time:

I don’t go in expecting things to change as quickly as probably I once did. So changing those realistic expectations and … remembering that and thinking *small steps* are steps in themselves. A client not even *cancelling* the next visit is a step in itself … it’s not *lowering* expectations … it’s being realistic about what *change is* for each client, especially the ones that have *bigger* obstacles and *higher* risk factors than most. (PHN 006)

For many of their clients, change was slow and rarely steady. The ability of PHNs to look for and see small changes, a skill that developed over time, was not only fulfilling but also a necessary indication that their work was having an impact. As one PHN expressed: “without *those* [small changes], the burnout would’ve probably happened years [ago]…there’s so much high-risk … you’re dealing with that if you don’t have a spark that, ‘oh my God am I making a difference?’ you can’t continue” (PHN 003).

### Challenges Related to Program Delivery

Although PHNs experienced a renewed sense of professionalism in delivering the NFP and highlighted the benefits of establishing strong therapeutic alliances with their clients – they also identified that a deeper engagement with clients meant a heightened level of connection and a potential for increased feelings of worrying about clients. Workload and organizational factors related to program structure were also highlighted as challenges to program delivery.

#### Personal impact of caring in the NFP

Beyond the challenge of meeting work obligations, PHNs also described experiencing a personal toll. When working with a population that faced near constant challenges, some PHNs struggled with maintaining their energy in the relationship. One PHN illustrated this by explaining:

There’s like a *constant flow* of bad stuff happening and it does, it does wear you down because you’re trying to be the *positive force* in these girls’ *lives*. That’s *really hard* to maintain that *intensity* and it takes… it’s *toll* itself physically, for myself emotionally. (PHN 007)

#### Worry and doubt

Many PHNs reported excessive thinking, worrying or even dreaming about their clients outside of work hours and particularly during times of client crisis. Even when PHNs attempted to create a boundary between work and home, they found it difficult to disengage from what they faced during the day.

While the completion of additional education on IPV gave PHNs much needed guidance and skills to engage with clients on this difficult topic, it also meant an increase in the amount of disclosures. For this PHN, once she started discussing IPV with clients, she was overwhelmed with the response:

Three people in one week disclosed *so much*. I was just like whoa, and I felt like the one girl I thought I can’t even leave here… I just want to put her in my car. I’m really *worried* for her, *worried.*… and then I went into the parking lot at Canadian Tire …had a little *cry*. It’s a cry for humanity …this is awful … I feel bad for these girls that live like this and somehow this passes for love. It’s pretty sad. (PHN 008)

When PHNs were sharing their concerns and worries about clients, they often reflected on their feelings of doubt within this relationship. For many, this was in the form of the question “did I do enough?” This PHN shared her experience of the difference between a “textbook” response and the often messy and painful reality of working in challenging and emotionally charged situations:

There were many times where I walked out of a supervision meeting or walked out of a home visit and I would sit in my car and cry, … when the woman tells you that her husband is beating her you need to call CAS [Child welfare agency] or this is what you do. It is very different when you’re actually *in the middle of observing* the violence or you’re in the middle of observing the woman telling you this is what happened to her … I’m supposed to say this now and so I’m going to ask her this question because that’s what the *tool tells me to ask her*. So you ask but you don’t always see a response from her in the way that the tool tells you they’re supposed to respond. They don’t respond that way. So you end up walking out of that visit and you bang your head against the steering wheel and you think oh my God did I say enough, did I say the right thing, did I do the right thing?… in my gut I just want to kidnap this baby, like I don’t know how to protect the baby. I don’t think I’m doing enough. I’m not good enough at this. (PHN 003)

#### Nothing left to give

The sense of “have I done enough” also surfaced beyond the context of decision-making. Many of the PHNs perceived a gap between what they felt they should be offering of themselves, and what they were actually able to offer. They described feelings of guilt around client’s outcomes, and struggled with taking responsibility for them. Home visits following crises were emotionally draining and PHNs reported needing to actively “work themselves up” as they felt they had “nothing left to give.” Rebuilding the relationship with the client took considerable energy and PHNs struggled with emotionally preparing for subsequent visits and the desire to be emotionally present for clients. This ongoing tension between work expectations and what the PHNs felt they could accomplish left them feeling overwhelmed and ultimately affected their ability to care.

#### Workplace Stress

Multiple factors were described as contributing to a sense of work stress and overload. Documentation, time spent in the car, accessing satellite offices, the scheduling demands of their clients and maintaining fidelity to the visiting schedule were all in competition for limited hours during the day

### Strategies to support PHNs’ NFP home visitation work

Within their practice, PHNs identified two primary strategies to manage the stresses and emotional responses arising from their work: 1) reflective supervision; and 2) informal peer debriefing.

#### Reflective supervision

PHNs described using reflective supervision sessions as an opportunity to seek advice on community resources, to ask questions around policies or procedures or to update the supervisor on the status of clients. There was consensus among the PHNs that the reflective practice sessions involved limited discussion of, or reflection on, the emotional experience of delivering the NFP. This PHN shared her perspective on reflective supervision:

I think a part of it was it’s very difficult to have a person you consult on cases with be your direct supervisor, the person that’s going to fill out your performance appraisal…I’m thinking a sane person shouldn’t be telling the supervisor some of the *struggles* that you might be feeling because it is a direct person you report to.(PHN 003)

Finding time for reflective supervision, and the availability of supervision following a difficult visit were additional limiting factors. As the nature of this relationship was vastly different from past experiences, it was suggested that both the PHNs and supervisor needed to adapt from previous patterns and learn the reflective process as practiced in the NFP. The relationship between the PHN and the supervisor, just like that of the PHN and the client, needed to be built over time for reflective practice to be effective.

#### Informal peer debriefing

PHNs disclosed that support was routinely received from fellow NFP team members first, partially as a result of their immediate availability, physical proximity in the office and their in-depth familiarity with the nature of the front-line experience. Debriefing with colleagues provided an important function of validation, as well as providing a learning opportunity for how to address a similar issue in the future. For some PHNs, debriefing with colleagues was a more effective, timely and comfortable way to engage in reflection. Informal debriefing with peers was described as an essential support and PHNs engaged in this practice on a daily basis, particularly as their colleagues really understood the nature of this work, that they “got it.”

## Discussion

This study is the first in-depth qualitative study to focus specifically on the professional and personal impacts on NFPs who engage in home visiting vulnerable families within the Canadian NFP context. Understanding the PHN experience in the NFP is a prerequisite to ensuring that PHNs are supported in this challenging and rewarding role. As the first step to the creation of an organizational culture that supports PHNs, the essence of the NFP intervention, an awareness of the impact on the PHN is essential.

The perceptions and experiences shared by PHNs indicated that the PHN-client relationship is at the core of home visiting. As the establishment and maintenance of this therapeutic relationship was fundamental to achieving successful maternal and child outcomes, the entire purpose of the NFP, supporting PHNs to engage with clients needs to be a priority. From an organizational perspective, the knowledge that NFP PHNs and supervisors will be personally impacted by the work that they do prioritizes the need for supports.

In this study, the PHN-client relationship was both a key source of satisfaction for PHNs and a source of personal stress. The development of a positive nurse-client relationship has been recognized as foundational to nursing [34] home visiting [6,14,35] and was identified as the most important outcome by vulnerable mothers [36]. In a Canadian study of PHNs evaluating PHN job satisfaction, PHNs identified that providing direct client care and making a difference were the aspects of work life that were the most satisfying [37]. While the focus of this study was not to examine the process of relationship development, analysis of the data revealed that the strategies NFP PHNs used to establish new relationships were consistent with published findings.

In an examination of how expert PHNs created relationships with vulnerable clients in a home visiting context, Zerwekh [38] suggested that novice PHNs, and the organizations they practiced in, tended to minimize the importance of the essential and time-consuming process of building and establishing the relationship and that the “real work” was achieving change. This was not the case in the NFP program as PHNs found the structure and philosophy of the NFP supportive to the enactment of these essential competencies.

Factors that decrease the PHNs ability to develop and maintain the often challenging relationships with clients will ultimately negatively influence program outcomes. One such factor is burnout, characterized by emotional exhaustion, depersonalization and a sense of inefficacy [39]. Within the organizational context, there are six factors that have been identified as antecedents that contribute to the development of burnout: workload, lack of control, lack of reward, lack of community, lack of fairness, and incongruence of values between the employee and the workplace [40]. Beyond the impact on the PHN-client relationship, burnout also contributes to negative work-related responses ranging from job dissatisfaction and decreased commitment to staff turnover [41,42]. Although burnout was described, it was not identified as a reason for any PHN leaving the program.

PHNs reported that workload was a major contributor to their stress. The complexity of client situations, combined with other administrative tasks, added to the workload burden of NFP nurses, even with a reduced caseload in Canada (20 versus a required caseload of 25 in the US) [23]. While this change was instituted to reflect differences in hours worked per week and annual number of vacation days, this reduction in caseload was thought to assist nurses in providing the level of care and support they desired and the program required. These findings support the work being done in the US to enhance the NFP model of care through the development of a strength and risk assessment framework allowing nurses to independently prioritize and adjust home visit frequency to support higher-risk families [43]. Strategies for prioritizing home visits and managing complex caseloads is an area for significant development in the NFP model.

Unlike burnout, compassion fatigue describes the emotional exhaustion that is a result of intense and prolonged contact with clients, the use of self, and exposure to stress [44]. Symptoms include lack of energy, apathy, poor judgment and indifference resulting in a desire to quit and an inability to care. In contrast to burnout, Boyle [45] suggested that rather than withdraw, those experiencing compassion fatigue responded by attempting to give even more of themselves to assist their clients until they are at the point at which they can no longer function. Those who were empathetic were at higher risk for compassion fatigue [46] and PHNs in this study were more likely to describe wanting to do more for their client than their role allowed rather than withdrawing from the relationship. It is important for PHNs and those who support them to be aware that both withdrawal and giving too much of one’s self are indicators of negative responses to their work and intervention or support may be necessary.

Client disclosures of current or past physical or sexual abuse, IPV or even exposure to the systematic impacts of poverty situations that place the PHN at high risk for vicarious trauma or secondary traumatic stress. Symptoms include: nightmares, intrusive images based on client’s stories or a sense that the world is no longer a safe place. While vicarious trauma or secondary traumatic stress has not been evaluated in PHN home visiting, it has been described by emergency room nurses caring for survivors of IPV [47] measured in hospital nurses [48] and in domestic violence advocates [49]. While all of these roles and contexts vary, there was the common element of exposure to a patient/client with a trauma history. NFP PHNs are at risk to experience secondary traumatic stress or vicarious trauma as clients in the NFP often had past or current issues with trauma.

Within this study, PHNs have described symptoms of burnout, compassion fatigue and vicarious trauma/secondary traumatic stress. These experiences were documented in other nursing practice settings [4] but it has not been well documented in home visiting. Further, there is not a clear understanding of how this experience may change over time or if there are predictable points at which PHNs may need additional support.

PHNs unanimously and frequently described the experience of worry for their clients. Worry was often linked to a sense of doubt of their effectiveness, particularly when client change was not observable. A sense of inefficacy is a contributor to burnout [40] and this needs to be openly acknowledged and discussed. Doubt was expressed by all PHNs in the study, most commonly by questioning responses to a given situation, asking themselves ‘what did I do wrong’ and taking client decisions personally. SmithBattle, Diekemper and Leander [50] in their study of the development of PHN clinical expertise noted that the tendency to want to “fix” client problems was common, particularly among less-experienced practitioners. This desire to fix leads to the inevitable realization that they cannot. The link between worry, doubt, “doing enough” and compassion fatigue has not been well described in the literature, although Kanter [51] suggested that creating realistic expectations serves a protective function. PHNs described this practice, in addition to a shift in how they defined success, as strategies to mitigate the uncertainties within their role.

While PHNs described challenges of working in the NFP, they also spoke of their experience of compassion satisfaction. The relationship with clients, personal growth and seeing the impact of their efforts were also reported as satisfying elements in a study of US PHNs [24]. There is limited research on factors contributing to compassion satisfaction among PHNs in home visiting, however organizational value alignment and length of time in the field were associated with compassion satisfaction in a study of domestic violence service providers [52]. Applying their finding to this study, PHNs reported a value alignment with the NFP program, however discrepancies or inconsistencies between the NFP model and expectations of the public health unit could have an impact on the PHNs’ compassion satisfaction. Increased experience may also be a factor in compassion satisfaction however further study is required to explore this.

PHNs described mitigating their sense of worry and work related stress through debriefing with colleagues, reflective supervision and recognizing that clients are responsible for their own decisions. PHNs in the initial pilot cohort had the experience of delivering the entire NFP program and graduating clients, whereas the newer PHNs had not yet reached this stage. With this experience, PHNs were able to reflect on the journey their clients had taken over time. This ability to see the broader perspective was indicative of gains in the PHN’s relational and perceptual skills [16] allowing them to see achievement over time. Literature supports that discussion with experienced colleagues assist with the development of perspective and in identification of over-involvement [14,50]. As the impact of worry and doubt may stem from the gap between theory, or program guidelines, and the unpredictability of the practice environment, this is a significant area where supervisors need to focus their reflective supervision goals [16].

The provision of weekly, individual clinical supervision meetings is a required element of the NFP program [53]. The PHNs described meeting with their supervisor as engaging in “reflective clinical supervision”, “an active and deliberate process when an individual is challenged and enabled to undertake the process of self-enquiry to empower the practitioner to realize desirable and effective practice within a reflexive spiral of personal transformation” (p.1405) [54]. Reflective supervision was similarly included in the American NFP program in the 1970’s as an opportunity to model theory to practice integration, promote professional development and address individual PHN’s concerns about their proficiency or lack of perceived progress with a client [55].

There is support for clinical supervision as a strategy to enhance program implementation [56], reduce nurse turnover [55] and compassion fatigue [51], support PHN role transition [56], reduce risk [57], identify problematic relationships with clients [58] and as a best practice to establish therapeutic relationships [34], however NFP PHNs reported a gap between the potential and actual gains of this process.

The supervisory experience can be limited by the lack of specialized training in reflective supervision methods [59]. Similarly, PHNs reported a ‘disconnect’ between emerging needs and their capacity to schedule supervision meetings [55]. In spite of these limitations, PHNs reported a growing capacity to examine their practice even in the absence of a problem [60] and the appreciated the value of dedicated individual sessions with their supervisor.

The importance of the supervisory role to the support and professional development of the PHN, the main intervention of the program, cannot be overstated. Organizations should ensure that support, including training and scheduling strategies, is routinely scheduled for supervisors, much as it is for PHNs. The NFP National Service Office (US organization that oversees NFP implementation) has developed online resources for supervisors as they have recognized the challenge of developing and maintaining supervisory skills in the reflective process [55]. In addition to increasing support in reflective supervision competencies, supervisors may benefit from engaging in direct practice alongside the front-line PHNs.

Of available supports, peer debriefing was perceived as the most valued and supportive strategy as well as the activity most consistently used by PHNs. Debriefing with others who “get it” was critically important as it provided PHNs validation for their actions and reactions from someone who understood their context. Maslach and Leiter [40] proposed that a lack of community was a necessary contributor to burnout, however the high level of functioning and support among this NFP team provided the PHNs with a strong sense of community in their workplace. This served a protective function against burnout and in turn fostered a sense of work engagement, thought to be the opposite of burnout [61].

The concept of the NFP team can also be considered from a broader scope. At any given time only five or six PHNs were active in the program and for the initial cohort, they were the first and only PHNs delivering the NFP in Canada. The community of support for Canadian PHNs was very small compared to the US, where the NFP is well established and an on-line community of NFP PHNs was available. PHNs in Canada expressed a desire to connect with a broader community to have additional resources and a forum to share experiences and ask questions and PHNs identified the benefits of the creation and fostering of a broader NFP community that would contribute to their work engagement [61].

### Study limitations

Multiple strategies were implemented in the conduct of this two-phase qualitative study to address frequent critiques of both qualitative secondary analyses and small descriptive studies, as well as to promote overall trustworthiness of the data. First, the implementation of a two-phase study design addressed the common critique that secondary analyses are limited in their ability to address a specific phenomenon in depth, if that issue was not the primary focus of the data [62]. In this study, the focus group data were purposefully analyzed to identify key themes that guided the development of the interview guide. This created a foundation for the second stage of the study, where original themes were explored in depth with a larger sample.

Although the sample size was small, this was the complete population of Ontario PHNs experienced in delivering the NFP and allowed for the in-depth exploration of multiple perspectives. However, this small pool within the specific context of a pilot site is a limitation. Within this group, over half were involved in the pilot project and were exposed to many challenges that come from being the first cohort. Considerable time and energy was spent adapting and learning program content without the benefit of on-site expertise and educating community partners about their role. Conversely, the remainder of the sample joined the NFP when education or support offered during the pilot study was no longer available. New PHNs joined one at a time benefiting from the support of experienced colleagues, but not from orienting with a peer group. These additional factors may have influenced the individual PHNs experience of work-related stress and may not be comparable to other settings. A study including a broader sample of PHNs across varying contexts is warranted. New sites within Canada are currently implemented within the context of a RCT underway in British Columbia. If the NFP is shown to be effective and new sites are developed, they will face similar issues of lack of local expertise and adapting content to fit their context.

## Conclusion

The fundamental component, and requirement, of the NFP is the PHN’s ability to form an empathic, therapeutic relationship with their client, yet it is this very relationship that also places the PHN at high risk for negative consequences. Workplace and organizational challenges contribute to the potential for PHNs to experience burnout. As the negative impact on the PHN increases, their ability to empathically connect with their client is impaired. Not only does this have potential repercussions for program outcomes and staff retention, but for professional and personal outcomes. To support PHNs, and the NFP program, approaches to address compassion fatigue, vicarious trauma/secondary traumatic stress and burnout should be considered. Appropriate supports and interventions are important to moderate the impact on PHNs. However, limits to the effectiveness of workplace interventions must be acknowledged. PHNs and supervisors require ongoing training for early recognition of situations where additional professional support is required.

The NFP has transformed the lives of not only the clients but the PHNs as well. As implementing NFP agencies, such as Hamilton Public Health Services, are required to deliver the NFP program with fidelity to the program model elements, the experiences of the Canadian PHNs documented in this study will be of high relevance to other NFP nurse home visitors working with clients in urban settings.

## Abbreviations

CAS: Children’s aid society
IPV: Intimate partner violence
NFP: Nurse-family partnership
PHN: Public health nurse
RCT: Randomized controlled trial
US: United States
